# An Explainable Machine Learning Framework for Predicting Hearing Aid Satisfaction: Integrating the HATASS Instrument and Clinical Insights

**DOI:** 10.3390/bioengineering13090985

**Published:** 2026-08-26

**Authors:** Seyma Arslanbas, Tahir Cetin Akinci, Ümit Can Çetinkaya, Sengul Terlemez

**Affiliations:** 1 Department of Audiology, Institute of Graduate Studies, Istanbul Aydin University, 34295 Istanbul, Türkiye; 2 Audiometry Program, Vocational School of Health Services, Istanbul Aydin University, 34295 Istanbul, Türkiye; 3 California Energy, Environment, and Technology (CE-CERT), Bourns College of Engineering, University of California Riverside, Riverside, CA 92507, USA; 4 Department of Audiology, Faculty of Health Sciences, Istanbul Aydin University, 34295 Istanbul, Türkiye; ucancetinkaya@aydin.edu.tr; 5 Independent Researcher, 34680 Istanbul, Türkiye; terlemezsengul@gmail.com

**Keywords:** hearing aid satisfaction, technology adaptation, explainable machine learning, audiological rehabilitation, feature importance, ensemble learning, user-centered care

## Abstract

Hearing aid technology adaptation and user satisfaction are influenced by multiple interacting demographic, clinical, and behavioral factors, making reliable prediction of outcomes challenging with conventional statistical approaches alone. This study proposes an explainable machine learning framework to investigate the multidimensional determinants of hearing aid satisfaction by integrating demographic characteristics, hearing aid-related variables, and patient-reported outcomes obtained from the Hearing Aid Technology Adaptation and Satisfaction Scale (HATASS). Five regression algorithms—Linear Regression, Decision Tree Regression (DTR), Random Forest Regression (RFR), Support Vector Regression, and Gradient Boosting Regression (GBR)—were comparatively evaluated using a five-fold cross-validation strategy. Predictive performance was assessed using the root mean square error (RMSE), mean absolute error (MAE), and the coefficient of determination ($R^{2}$), while model interpretability was investigated through cross-validated out-of-bag permutation feature importance analysis. Among the evaluated algorithms, Random Forest Regression achieved the most consistent predictive performance, yielding the lowest average RMSE (14.225) and the highest average $R^{2}$ (0.146) under the adopted validation framework. Although the overall predictive performance remained modest, the explainability analysis consistently identified age as the most influential predictor, followed by onset year, education level, hearing aid usage duration, and daily hearing aid use. In contrast, gender, battery type, tinnitus, and vertigo contributed comparatively less to model predictions. These findings indicate that hearing aid adaptation and satisfaction arise from complex nonlinear interactions among demographic, behavioral, clinical, and device-related characteristics rather than isolated linear associations. The proposed framework provides an interpretable, internally validated analytical approach for investigating hearing aid technology adaptation and satisfaction and establishes a foundation for future studies that integrate comprehensive audiological measurements, longitudinal follow-up data, and independent external validation to support the development of more transparent and personalized hearing healthcare systems.

## 1. Introduction

Hearing loss is a health condition that requires a multidisciplinary approach and negatively impacts many areas of life, from cognitive function to social participation. Current data predict that the global burden of hearing loss is increasing, and by 2050, approximately 2.5 billion individuals will experience varying degrees of hearing loss, with more than 700 million requiring rehabilitation [1]. Hearing aids are among the most commonly prescribed rehabilitation devices for individuals with sensorineural hearing loss. Although significant advancements have been made in hearing aid technology over the past decade, including smartphone integration, wireless connectivity, and artificial intelligence [2,3], many users continue to report difficulties with sound quality, device comfort, and adaptation to hearing aids. These difficulties directly influence hearing aid acceptance, long-term use, and, consequently, rehabilitation success.

Systematic reviews published in recent years have identified numerous determinants of hearing aid use and satisfaction. These studies demonstrate that factors such as hearing sensitivity, self-perceived hearing difficulties, social support, educational level, and economic conditions are not merely passive variables but dynamic determinants that modulate the level of hearing aid use in both positive and negative directions [4]. Daily communication difficulties, prior hearing aid experience, and advanced hearing aid technologies have been identified as key factors in successful hearing aid adaptation and satisfaction [5,6]. However, identifying the underlying drivers of these individual-specific difficulties remains a challenge, as traditional clinical assessment tools often fail to capture the complexity of user satisfaction in the era of AI-integrated hearing technology. Furthermore, when analyzing these multidimensional hearing aid outcomes, previous studies have predominantly relied on traditional statistical analyses, descriptive evaluations, or questionnaire-based interpretations. Traditional statistical methods generally rely on linear assumptions and may be insufficient to capture the complex, multidimensional interactions that influence hearing aid satisfaction [7].

Recent advancements in machine learning have provided new opportunities to complement traditional statistical analyses by modeling complex, nonlinear relationships and supporting predictive healthcare analytics [8]. Algorithms such as ensemble learning, Support Vector Machines (SVMs), and Artificial Neural Networks (ANNs) have demonstrated robust performance across various predictive models in the audiology literature [9,10]. In particular, the use of predictive algorithms is steadily increasing in the development of Clinical Decision Support Systems (CDSSs) and the prognostication of rehabilitation outcomes [11,12]. These technological advancements provide additional opportunities to analyze the multidimensional, complex nature of audiological data and may complement conventional clinical assessment approaches by supporting more individualized, data-informed hearing healthcare.

However, in hearing aid rehabilitation, merely achieving high predictive accuracy is insufficient for models to be successfully translated into clinical practice. For clinicians to trust a decision support system, algorithms must move beyond their “black-box” nature; the results generated should be interpretable and transparent and provide clinically relevant insights into how different variables are associated with user satisfaction [5,13]. In this context, explainable artificial intelligence approaches represent a critical research area for audiology-related healthcare applications and for reliable clinical decision-making.

Motivated by these limitations in the existing literature, the present study proposes an explainable machine learning framework to investigate hearing aid technology adaptation and satisfaction, using demographic, clinical, and hearing aid-related variables collected from adult hearing aid users. The framework integrates user-reported outcomes obtained from the HATASS, a multidimensional instrument recently developed and psychometrically validated by the authors [14], with routinely collected demographic and hearing aid-related characteristics. Five regression-based machine learning algorithms, namely Linear Regression (LR), Decision Tree Regression (DTR), Random Forest Regression (RFR), Support Vector Regression (SVR), and Gradient Boosting Regression (GBR), were comparatively evaluated using a five-fold cross-validation framework to investigate their ability to capture potentially complex nonlinear relationships among the investigated variables. In addition to comparative predictive performance evaluation, cross-validated out-of-bag permutation feature importance analysis was performed within the Random Forest model to quantify the relative contribution of individual predictors and improve model interpretability.

Unlike many previous studies in audiology that primarily rely on descriptive statistical analyses or conventional inferential approaches, this study investigates the feasibility of combining explainable machine learning with transparent model interpretation to better understand the multidimensional factors associated with the adaptation to and satisfaction with hearing aid technology. Rather than establishing causal relationships or proposing an autonomous clinical decision-making system, the proposed framework is intended as an interpretable analytical approach to exploring complex associations among demographic, behavioral, clinical, and hearing-aid-related variables. By integrating comparative machine learning with cross-validated explainability analysis, this study provides a reproducible computational framework that may support future research on personalized audiological rehabilitation and facilitate the development of more transparent, data-informed analytical tools following validation in larger, independent, and longitudinal clinical datasets.

## 2. Materials and Methods

This section describes the methodological framework adopted in the present study, including the study design, dataset characteristics, data preprocessing procedures, machine learning methodology, model validation strategy, and explainability analysis. Particular emphasis is placed on methodological transparency, reproducibility, and the systematic comparison of multiple supervised regression algorithms within a unified analytical framework. The proposed workflow was designed to provide an interpretable and reproducible approach for investigating the multidimensional factors associated with hearing aid technology adaptation and satisfaction.

### 2.1. Study Design and Participants

This study was conducted as a cross-sectional secondary analysis of previously collected data from adult hearing aid users. The original dataset was collected under institutional ethical approval granted by the Istanbul Aydin University Non-Interventional Clinical Research Ethics Committee (Date: 11 December 2024, Decision No. 123/2024), and written informed consent was obtained from all participants during the original data collection. The present secondary analysis was performed in accordance with the principles of the Declaration of Helsinki and received approval from the Non-Interventional Clinical Research Ethics Committee of Istanbul Aydin University (Date: 16 July 2026, Decision No. 287/2026).

The dataset consisted of 583 adult hearing aid users with heterogeneous demographic characteristics, hearing aid experience, and device-related features. Participants had a mean age of 53.3 years (SD = 17.0), ranging from 18 to 87 years, as summarized in Table 1. This heterogeneous study population enabled the investigation of hearing aid technology adaptation and satisfaction across a broad spectrum of adult hearing aid users.

The primary objective of this study was to develop and evaluate an explainable machine learning framework to investigate hearing aid technology adaptation and satisfaction, using demographic, clinical, and hearing aid-related variables to predict the total HATASS score. Rather than relying exclusively on conventional statistical analyses, the proposed framework comparatively evaluated multiple regression-based machine learning algorithms within a five-fold cross-validation framework and complemented predictive performance evaluation with explainability analysis to investigate the complex relationships underlying hearing aid technology adaptation and satisfaction.

### 2.2. Dataset and Variables

The dataset comprised demographic characteristics, hearing aid-related information, and multidimensional questionnaire responses collected from adult hearing aid users. The predictor variables included age, gender, onset year of hearing loss, tinnitus status, vertigo status, hearing aid usage duration, daily hearing aid use, educational level, and hearing aid power supply type (disposable battery or rechargeable). These variables were selected because they represent routinely collected demographic, clinical, behavioral, and device-related characteristics that may be associated with hearing aid technology adaptation and user satisfaction.

The target variable of the proposed predictive framework was the total HATASS score. The HATASS is a multidimensional instrument developed and psychometrically validated in Turkish by Arslanbas et al. To facilitate international accessibility, an unvalidated English-language reference version of the instrument is provided in the Supplementary Materials. The instrument consists of three principal dimensions:

- Technological satisfaction experience (TSE);
- Digital functionality (DF);
- Usage difficulty (UD).

The total HATASS score was analyzed as a continuous outcome variable throughout the predictive modeling framework. Higher HATASS scores indicate greater technology adaptation and user satisfaction. The HATASS consists of 29 items rated on a five-point Likert scale, resulting in a theoretical total score range of 29–145. Since no validated categorical cut-off values (e.g., low, moderate, or high satisfaction) have been established for the instrument, the total score was modeled as a continuous variable rather than categorized into predefined satisfaction groups.

The psychometric properties of the HATASS, including internal consistency, factor structure, and reliability coefficients, are summarized in the Supplementary Materials to document the instrument’s validity and reliability. To prevent data leakage during model development, individual HATASS items and subscale scores were excluded from the predictor matrix and were not used as independent variables. Only the total HATASS score served as the prediction target. Furthermore, all data transformations that require parameter estimation, such as numerical feature standardization, were estimated exclusively from the training data within each cross-validation fold and then applied to the corresponding validation data.

Comprehensive psychometric validation details, including exploratory and confirmatory factor analyses, convergent and discriminant validity, internal consistency, and stability analyses, are provided in the Supplementary Materials (Tables S1–S12).

### 2.3. Data Preprocessing

Data preprocessing was performed prior to predictive modeling to improve data consistency and analytical reliability. Initially, the dataset was inspected in MATLAB R2025b to identify missing values and verify the integrity of the selected predictor variables and the target HATASS scores. No missing values were identified in the variables included in the analysis.

Negatively worded questionnaire items were reverse-coded to ensure directional consistency throughout the HATASS instrument. Reverse coding was performed according to Equation (1):

(1) $$x_{rev}=6-x$$

where *x* denotes the original Likert-scale response and $x_{rev}$ represents the reverse-coded score. This transformation ensured that higher item scores consistently reflected greater hearing aid technology adaptation and user satisfaction.

Following reverse coding, the total HATASS score was calculated by summing the positively coded and reverse-coded questionnaire items. The resulting total score served as the continuous target variable throughout all predictive modeling analyses. Prior to model development, categorical predictors were encoded using MATLAB’s categorical data representation, whereas numerical predictors were standardized within each training partition to prevent information leakage during cross-validation. Descriptive statistical analyses were subsequently performed to summarize participant characteristics, examine score distributions, and characterize the overall variability in hearing aid technology adaptation and satisfaction across the study population.

### 2.4. Machine Learning Framework

The proposed framework employed supervised regression-based machine learning algorithms to investigate hearing aid technology adaptation and satisfaction by predicting the total HATASS score from demographic, clinical, behavioral, and hearing aid-related variables. The predictor matrix was constructed using routinely collected participant characteristics, whereas the target vector consisted of the corresponding total HATASS scores. The selected regression models represent complementary linear, tree-based, ensemble, and kernel-based learning paradigms, thereby enabling a systematic comparison of different predictive strategies within a unified preprocessing and validation framework.

The predictor matrix is represented as

(2) $$X=[x_{1},x_{2},x_{3},\ldots ,x_{n}]$$

where *X* contains the predictor variables, including age, gender, onset year of hearing loss, tinnitus status, vertigo status, hearing aid usage duration, daily hearing aid use, educational level, and hearing aid power supply type.

The regression target variable is represented as

(3) $$Y=[y_{1},y_{2},y_{3},\ldots ,y_{n}]$$

where *Y* denotes the participant-specific total HATASS scores. As defined in Equations (2) and (3), the proposed machine learning framework predicts hearing aid technology adaptation and satisfaction using the available demographic, clinical, behavioral, and hearing aid-related characteristics.

To comparatively evaluate predictive performance, five supervised regression algorithms were implemented:

- Linear Regression (LR);
- Decision Tree Regression (DTR);
- Random Forest Regression (RFR);
- Gradient Boosting Regression (GBR);
- Support Vector Regression (SVR).

These algorithms were selected because they represent widely used regression techniques with distinct learning mechanisms, enabling comparisons among linear, tree-based, ensemble, and kernel-based approaches under identical preprocessing conditions.

To obtain a robust estimate of model generalization, predictive performance was evaluated using five-fold cross-validation. The complete dataset was randomly partitioned into five approximately equal subsets. During each iteration, four folds were used for model training, and one fold was reserved for validation. This procedure was repeated five times so that every observation served once as validation data. Performance metrics were computed independently for each validation fold and subsequently averaged across the five folds to obtain the final model performance estimates.

To improve methodological transparency and facilitate reproducibility, the complete analytical workflow adopted in this study is summarized in Algorithm 1. The pseudocode describes the entire analytical pipeline, including data preprocessing, cross-validation, model training, prediction, performance evaluation, and cross-validated permutation feature importance analysis.
**Algorithm 1** Cross-Validated Machine Learning Workflow**Require:** Predictor matrix *X* and target vector *Y***Ensure:** Cross-validated performance metrics and ranked predictor importance  1:Load study dataset  2:Extract predictor matrix *X* and target vector *Y*  3:Reverse-code negatively worded HATASS items  4:Generate five-fold cross-validation partitions  5:**for** each validation fold **do**  6:    Split data into training and validation subsets  7:    Fit preprocessing pipeline on the training subset  8:    Transform the validation subset using the fitted preprocessing  9:    **for** each model M∈{LR,DTR,RFR,GBR,SVR} **do**10:        Train model *M*11:        Predict HATASS scores for the validation subset12:        Compute RMSE, MAE, and $R^{2}$13:    **end for**14:    Compute OOB permutation importance using the RFR model15:**end for**16:Average RMSE, MAE, and $R^{2}$ across all folds17:Average permutation importance across all folds18:Rank predictors according to mean permutation importance19:Compare the predictive performance across all models

### 2.5. Hyperparameter Selection

All regression models were implemented using the built-in machine learning functions available in MATLAB R2025b. Unless otherwise specified, the default hyperparameter settings provided by the software were adopted for all investigated regression models. This strategy ensured a fair and consistent comparison among LR, DTR, RFR, GBR, and SVR under identical experimental conditions.

No systematic hyperparameter optimization, such as grid search, randomized search, or Bayesian optimization, was performed. The primary objective of the present study was to comparatively evaluate the predictive behavior, generalization performance, and interpretability of widely used regression algorithms rather than to maximize predictive accuracy through model-specific parameter tuning. Consequently, all models were evaluated using a common experimental configuration, providing a transparent, reproducible, and unbiased comparative framework.

### 2.6. Performance Evaluation

The predictive performance of the investigated machine learning models was evaluated using RMSE, MAE, and the coefficient of determination ($R^{2}$). These complementary performance metrics were selected to quantify prediction accuracy, average prediction error, and explanatory capability across the evaluated regression models. Model performance was estimated using five-fold cross-validation, and the reported RMSE, MAE, and $R^{2}$ values correspond to the average performance obtained across the five validation folds.

The RMSE metric is defined as

(4) $$RMSE=\sqrt{\frac{1}{n}\sum_{i=1}^{n}{\left(y_{i}-{\hat{y}}_{i}\right)}^{2}}$$

where $y_{i}$ denotes the observed HATASS score and ${\hat{y}}_{i}$ represents the corresponding predicted score. As shown in Equation (4), RMSE penalizes larger prediction errors more heavily because of the squared-error formulation, making it particularly sensitive to substantial deviations between predicted and observed values.

The MAE metric is expressed as

(5) $$MAE=\frac{1}{n}\sum_{i=1}^{n}\left|y_{i}-{\hat{y}}_{i}\right|$$

As defined in Equation (5), MAE measures the average magnitude of prediction errors without disproportionately emphasizing extreme deviations. Consequently, it provides an intuitive estimate of the average prediction error across all validation samples.

The coefficient of determination is calculated as

(6) $$R^{2}=1-\frac{\sum_{i=1}^{n}{\left(y_{i}-{\hat{y}}_{i}\right)}^{2}}{\sum_{i=1}^{n}{\left(y_{i}-\bar{y}\right)}^{2}}$$

where $\bar{y}$ denotes the mean observed HATASS score. As defined in Equation (6), the coefficient of determination quantifies the proportion of variability in the observed outcomes explained by the regression model. Unlike RMSE and MAE, which quantify prediction error, $R^{2}$ reflects explanatory capability, with larger values indicating greater agreement between predicted and observed outcomes.

### 2.7. Feature Importance and Explainability Analysis

To improve model interpretability, an explainability analysis was performed using the RFR model. Predictor importance was quantified using out-of-bag (OOB) permutation feature importance within the five-fold cross-validation framework. For each validation fold, permutation importance scores were estimated from the corresponding Random Forest model, and the mean and standard deviation across the five folds were calculated to obtain a robust estimate of predictor importance.

Permutation feature importance evaluates the contribution of each predictor by measuring the reduction in model performance when the values of a single variable are randomly permuted, while all other predictors remain unchanged. Predictors that produce greater reductions in model performance after permutation are considered more influential.

The resulting mean permutation importance values were used to rank demographic, clinical, behavioral, and hearing aid-related variables according to their relative contribution to predicting total HATASS scores. Because the importance estimates were averaged across multiple validation folds, the proposed approach reduces the influence of a single data partition and provides a more stable assessment of predictor relevance.

It should be emphasized that permutation importance quantifies the contribution of individual variables to the predictive model rather than statistical significance or causal relationships. Consequently, the proposed explainability framework complements predictive performance evaluation by providing an interpretable description of model behavior while preserving clinician-centered decision-making and supporting future research on hearing aid technology adaptation and satisfaction.

## 3. Results

This section presents the statistical analyses, explainability investigations, and machine-learning-based predictive results obtained from the dataset under investigation. Initially, descriptive statistical analyses were conducted to evaluate the demographic structure of the participant population together with the distributional characteristics of the total HATASS scores. Subsequently, correlation-based analyses were performed to examine linear relationships among demographic, clinical, and hearing aid-related variables.

Following the statistical evaluation stage, multiple regression-based machine learning algorithms were compared to assess predictive performance and potentially nonlinear relationships between adaptation and satisfaction outcomes. In addition, a feature importance analysis and an explainability-oriented evaluation were conducted to identify the variables that contributed most substantially to the predictive behavior within the proposed framework.

### 3.1. Descriptive Statistical Analysis

Descriptive statistical analyses were initially conducted to investigate the dataset’s demographic structure and the distributional characteristics of the total HATASS scores. The study population consisted of 583 hearing aid users with a relatively balanced gender distribution. Male participants represented 53.34% of the dataset, while female participants accounted for 46.66%. The average participant age was 53.31 years with a standard deviation of 16.97 years, indicating that the dataset included a broad adult population with heterogeneous demographic characteristics.

The total HATASS score indicated relatively high levels of technology adaptation and satisfaction among the participants. The mean HATASS score was 127.79 with a standard deviation of 15.55, while the observed minimum and maximum values were 49 and 145, respectively. These findings indicate that the dataset preserved measurable variability despite the overall concentration toward higher levels of adaptation and satisfaction. Table 1 summarizes the demographic and score-related descriptive statistics obtained from the investigated dataset.

**Figure 1:**
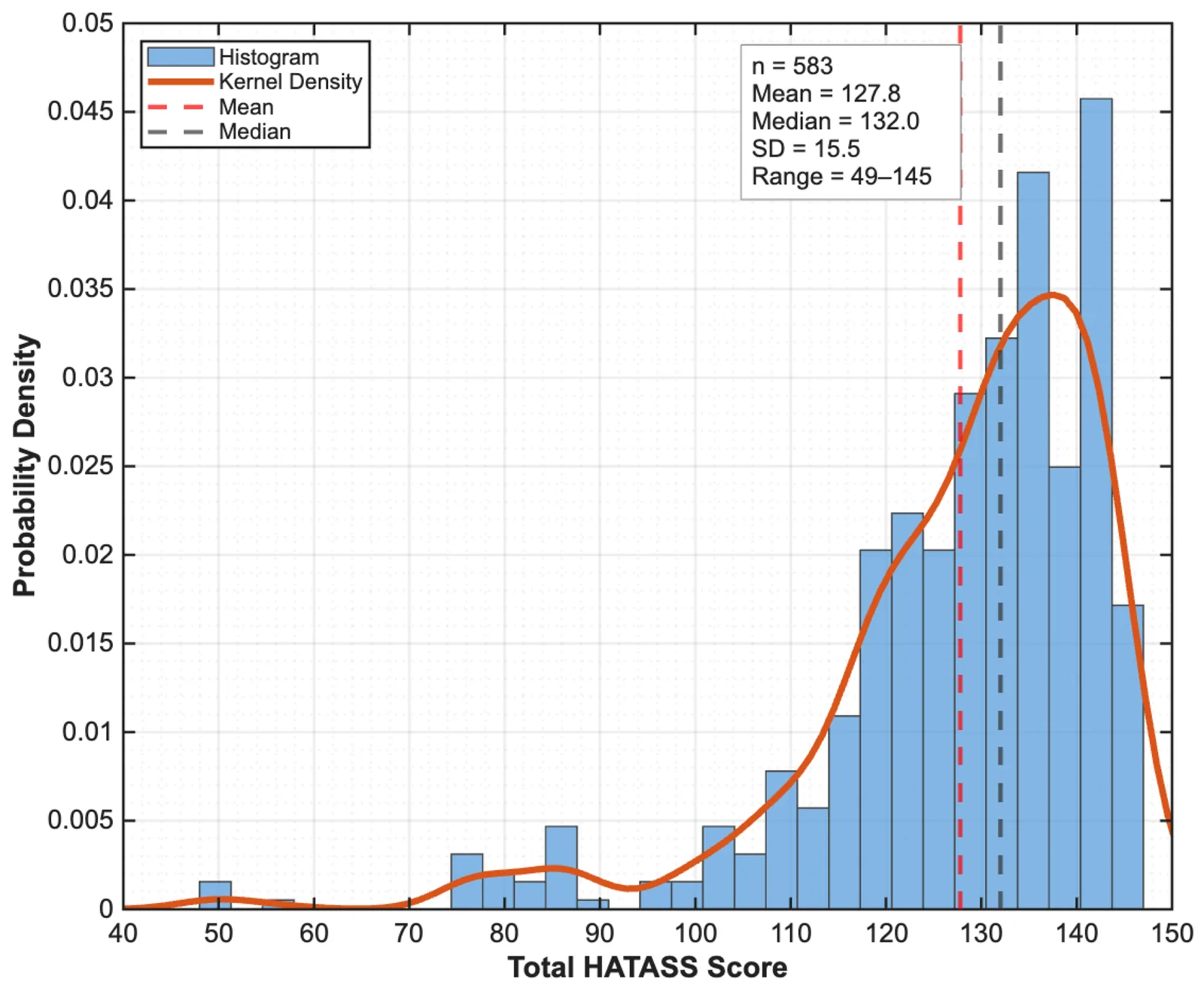
Distribution of the total HATASS scores in the study cohort. The histogram is overlaid with a kernel density estimate, and the dashed vertical lines indicate the sample mean and median. The distribution shows a concentration of observations toward the higher end of the HATASS score range while retaining sufficient variability for subsequent regression-based machine learning analyses.

The distribution of the total HATASS scores was examined to characterize the overall variability of the outcome variable prior to predictive modeling. Figure 1 presents the score distribution using a histogram overlaid with a kernel density estimate, providing a visual assessment of the distributional characteristics of the investigated dataset.

As illustrated in Figure 1, the majority of participants exhibited relatively high HATASS scores, although substantial variability remained across the investigated cohort. The observed distribution indicates that technology adaptation and satisfaction outcomes were not uniformly distributed among participants, supporting the dataset’s suitability for regression-based predictive modeling and reflecting the multidimensional nature of hearing aid technology adaptation and satisfaction.

### 3.2. Correlation Analysis

A Pearson correlation analysis was performed to investigate the linear relationships among the demographic, clinical, and hearing aid-related variables included in the present dataset. The analysis indicated that participant age exhibited a negligible linear association with the total HATASS score. The corresponding Pearson correlation coefficient was calculated as

(7) $$r_{\mathrm{Age},\mathrm{HATASS}}=0.0062$$

As shown in Equation (7), the linear relationship between participant age and the total HATASS score was essentially negligible. This finding suggests that age alone provides limited linear information for explaining adaptation to and satisfaction with hearing aid technology in the investigated cohort. Although previous studies have frequently associated increasing age with reduced technology adaptation or perceived usability, the present results indicate that hearing aid satisfaction is more likely to depend on complex interactions among demographic, behavioral, clinical, and hearing aid-related factors than on age considered as an isolated predictor.

**Figure 2:**
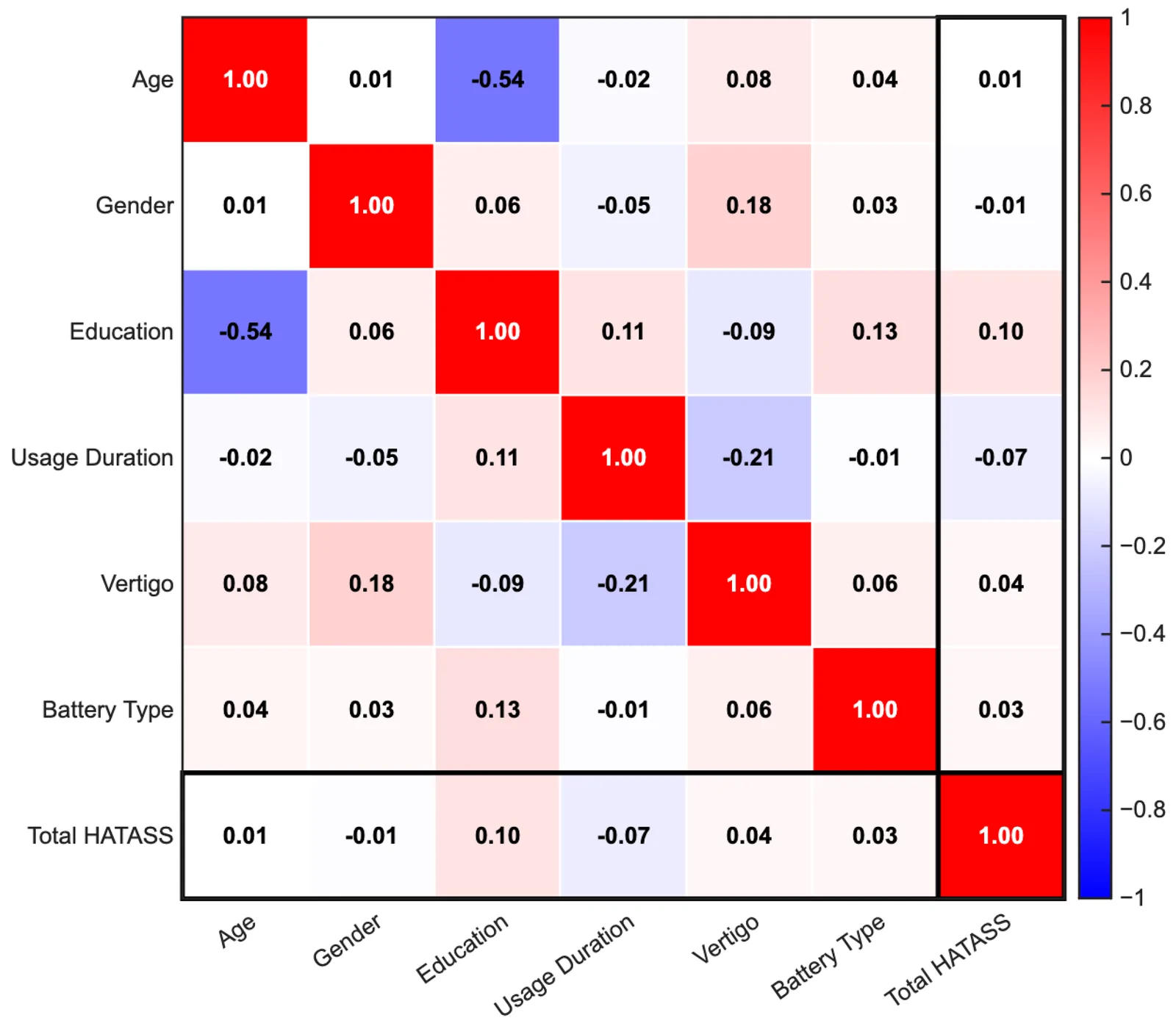
Correlation matrix illustrating the relationships among the demographic, clinical, and hearing aid-related variables included in the present dataset. Most of these variables demonstrated relatively weak direct linear correlations with the total HATASS scores, supporting the use of nonlinear machine learning approaches for predictive modeling.

To further examine the relationships among the investigated variables, Pearson correlation coefficients were calculated for the demographic, clinical, hearing aid-related, and outcome variables. The resulting correlation matrix is presented in Figure 2.

As shown in Figure 2, the total HATASS score exhibited only negligible to weak pairwise linear correlations with the investigated predictor variables. In contrast, the strongest correlation observed in the matrix was between age and educational level (r=−0.54), whereas the remaining pairwise correlations were generally weak. These findings indicate that individual demographic or hearing aid-related variables alone provide limited linear information for explaining variation in technology adaptation and satisfaction.

The overall correlation pattern, therefore, supports the use of nonlinear machine learning approaches to investigate relationships that may not be captured by conventional linear statistical analyses. Rather than indicating that individual variables are unimportant, the weak pairwise correlations suggest that technology adaptation and satisfaction are likely influenced by interactions among multiple demographic, clinical, behavioral, and hearing aid-related factors, which were subsequently explored using regression-based machine learning models.

### 3.3. Predictive Performance of Machine Learning Models

Regression-based machine learning models were compared for predicting the total HATASS score using demographic, clinical, and hearing aid-related variables. The evaluated algorithms included LR, DTR, RFR, SVR, and GBR. Model performance was assessed using five-fold cross-validation to obtain a more reliable estimate of predictive performance across different training and validation subsets. Prediction accuracy was evaluated using the RMSE, MAE, and coefficient of determination ($R^{2}$). The average cross-validation results are summarized in Table 2.

The cross-validation results revealed measurable differences among the investigated regression algorithms. Random Forest Regression achieved the lowest average RMSE (14.228) together with the highest average coefficient of determination ($R^{2}=0.146$), indicating the most consistent overall predictive performance across the validation folds. Support Vector Regression produced the lowest average MAE (9.758), whereas Decision Tree Regression and Gradient Boosting Regression yielded higher prediction errors and negative average $R^{2}$ values, indicating limited generalization under the adopted validation framework. The relatively low $R^{2}$ values observed across all evaluated models suggest that the demographic, clinical, and hearing aid-related variables included in the present study explain only a modest proportion of the variability in total HATASS scores. These findings indicate that hearing aid technology adaptation and satisfaction are influenced by additional multidimensional factors that were not represented in the current dataset.

**Figure 3:**
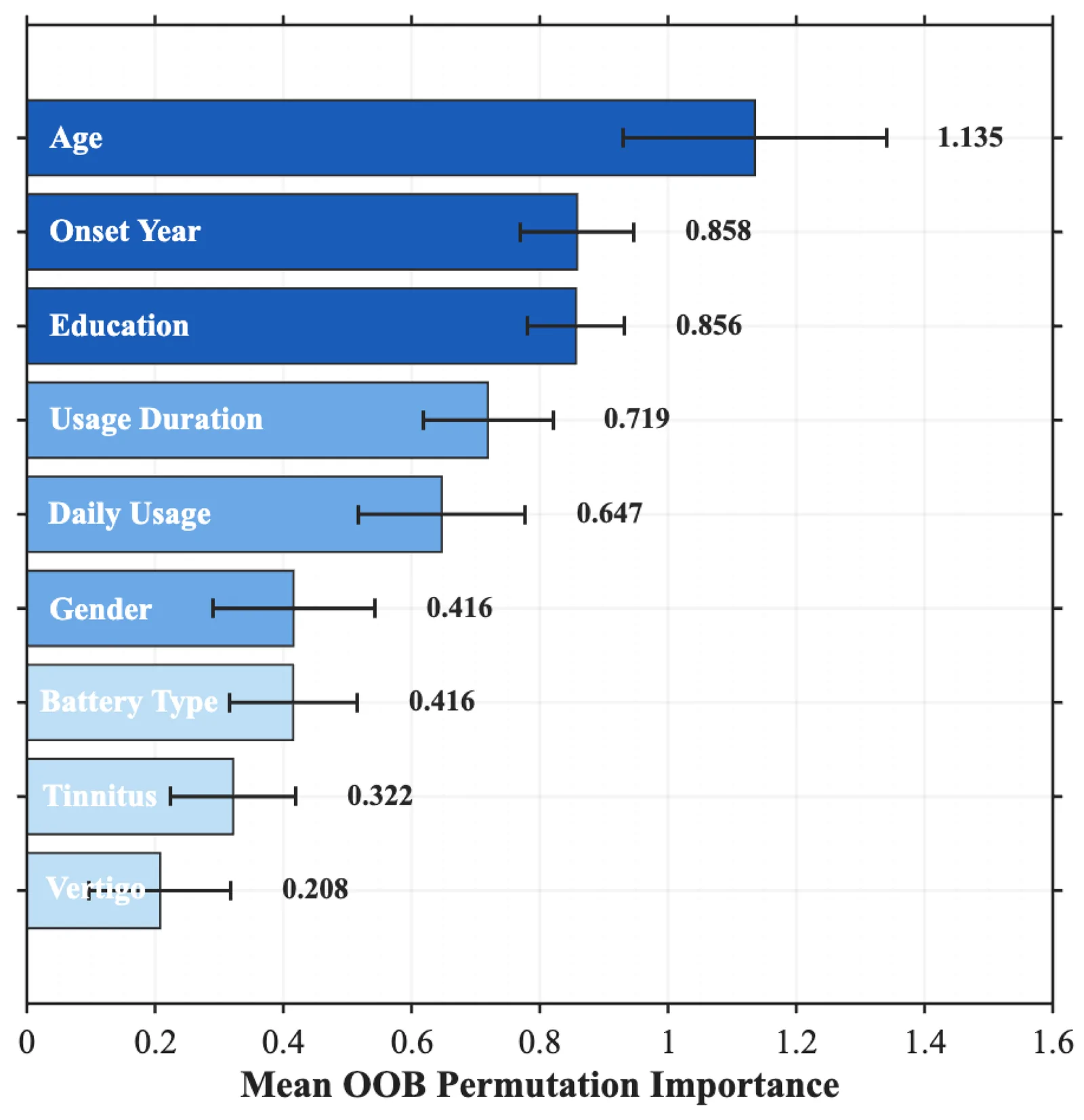
Cross-validated permutation feature importance obtained from the Random Forest Regression model. Bars represent the mean out-of-bag (OOB) permutation importance averaged across the five cross-validation folds, whereas error bars indicate one standard deviation. Larger importance values correspond to greater contributions of individual predictors to the prediction of total HATASS scores. Age, onset year of hearing loss, and education level were identified as the most influential predictors, whereas tinnitus and vertigo contributed comparatively less to model predictions.

To improve model interpretability, permutation-based feature importance analysis was subsequently performed using the Random Forest Regression model. Figure 3 presents the mean out-of-bag (OOB) permutation importance of each predictor averaged across the five-fold cross-validation procedure, together with one standard deviation illustrating the variability of feature importance estimates across independently trained models.

As shown in Figure 3, age was identified as the most influential predictor, followed by the onset year of hearing loss and education level. Hearing aid usage duration and daily hearing aid use also contributed substantially to the model predictions, whereas gender, battery type, tinnitus, and vertigo exhibited comparatively lower importance values. These findings indicate that demographic characteristics, together with hearing-loss history and behavioral variables, contributed more strongly to the prediction of total HATASS scores than the remaining variables considered in the present study.

**Figure 4:**
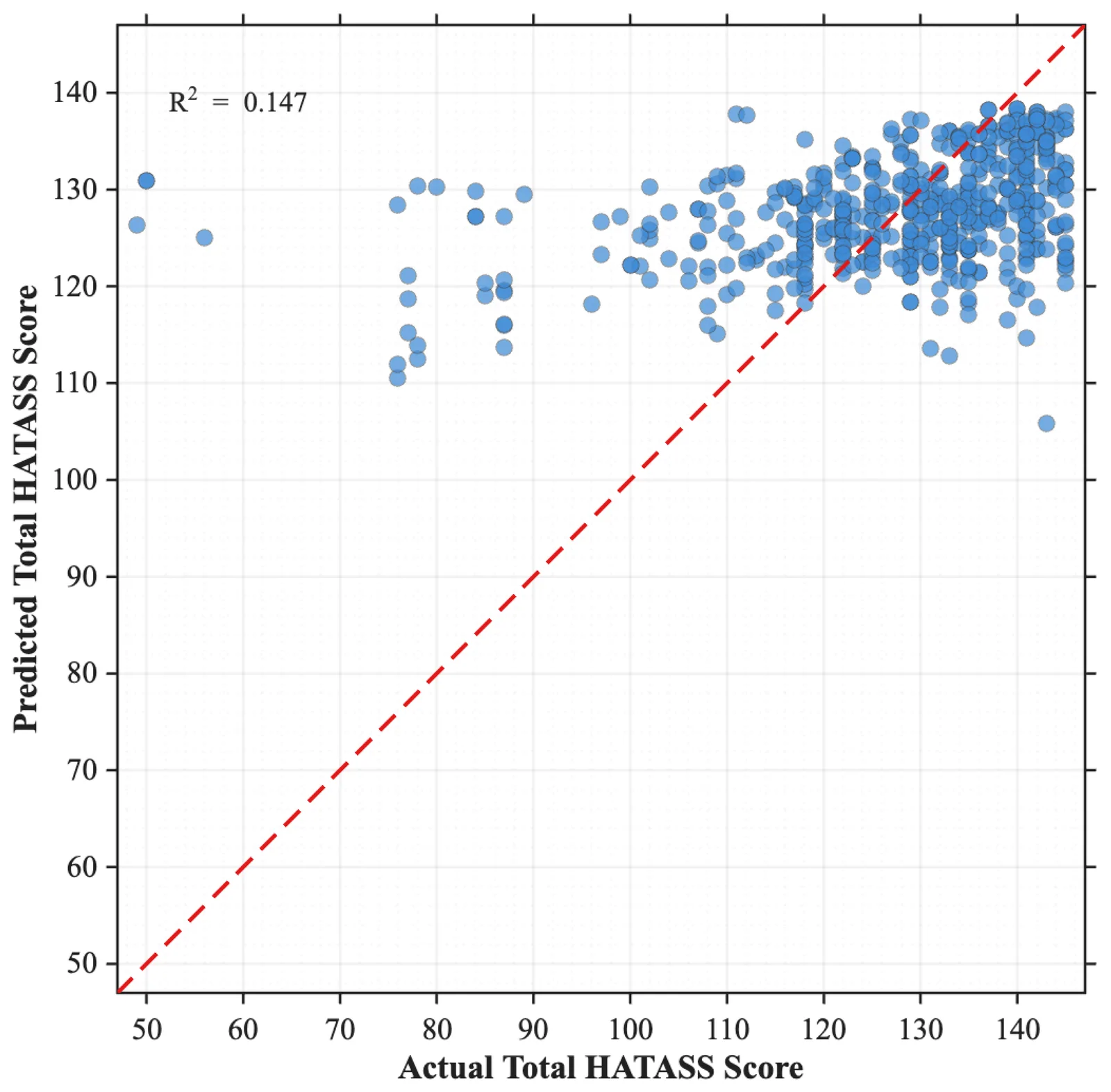
Cross-validated actual versus predicted total HATASS scores obtained using the Random Forest Regression model. The dashed red line represents perfect agreement (y=x). The figure illustrates the relationship between observed and predicted HATASS scores across the five-fold cross-validation procedure.

Figure 4 further illustrates the predictive behavior of the Random Forest Regression model. Most observations followed the general trend defined by the line of perfect agreement, although noticeable dispersion remained across the range of HATASS scores. Prediction errors were more evident for participants with relatively low HATASS scores, whereas predictions for moderate-to-high scores exhibited comparatively better agreement. The tendency of predicted values to cluster in the middle and upper score ranges is consistent with the modest predictive performance observed during cross-validation. Together, the findings presented in Table 2 and Figure 3 and Figure 4 indicate that Random Forest Regression provided the most consistent overall predictive performance among the investigated algorithms while simultaneously offering an interpretable framework for identifying the variables that contributed most strongly to hearing aid technology adaptation and satisfaction.

**Figure 5:**
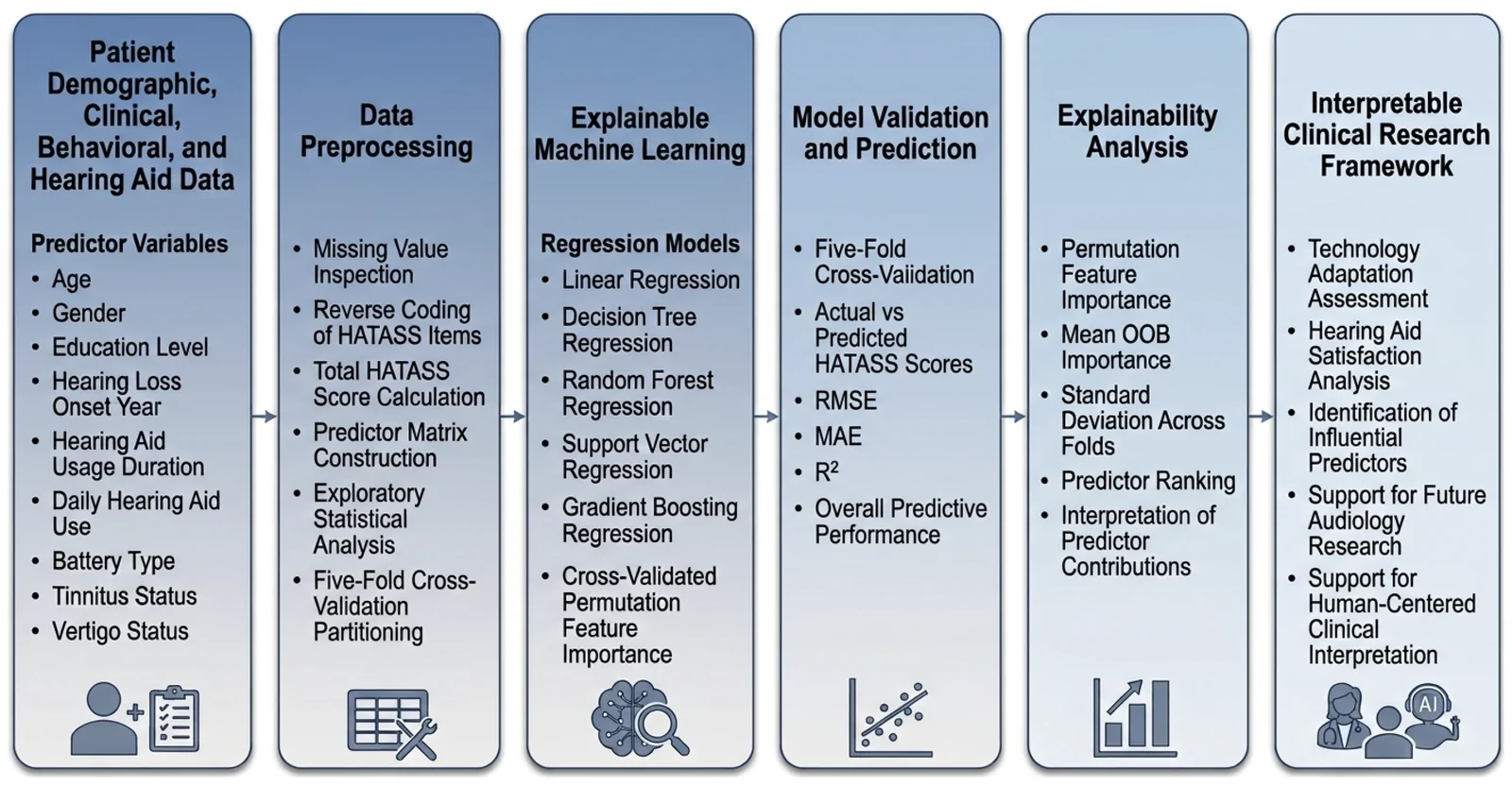
Conceptual explainable machine learning framework for predicting the total HATASS score from demographic, clinical, behavioral, and hearing aid-related variables. The framework summarizes the proposed analytical workflow by integrating data preprocessing, interpretable machine learning, predictive assessment, and clinician-oriented interpretation to support future audiology research.

Based on the predictive performance evaluation and the cross-validated permutation feature importance analysis, a conceptual explainable machine learning framework was developed to summarize the overall analytical workflow of the proposed approach (Figure 5). The framework illustrates how routinely collected demographic, clinical, behavioral, and hearing aid-related variables can be integrated with interpretable machine learning models to predict the total HATASS score and facilitate transparent interpretation of model outputs. Rather than representing a deployable clinical decision support system, the proposed framework should be regarded as an interpretable analytical workflow intended to support future research on user-centered audiological rehabilitation and hearing aid technology adaptation and satisfaction.

## 4. Discussion

The findings of this study demonstrate the potential of explainable machine learning to improve the interpretation of factors associated with hearing aid technology adaptation and satisfaction. Consistent with the growing interest in predictive algorithms and explainable artificial intelligence for audiological applications and CDSS [11,12], the proposed framework illustrates how demographic, clinical, and hearing aid-related variables can be integrated with user-reported outcome measures to support predictive assessment. A major strength of the proposed approach is the integration of the HATASS questionnaire with routinely collected demographic and clinical variables, enabling hearing aid satisfaction to be investigated as a multidimensional construct rather than as a function of isolated user characteristics. By combining predictive modeling with explainability analysis, the proposed framework provides an interpretable analytical framework for investigating relationships between objective clinical variables and subjective user experiences [5,12].

The negligible linear association observed between age and the total HATASS score during the initial statistical analyses might traditionally suggest that age has limited predictive value. However, within the nonlinear Random Forest Regression model, age consistently exhibited the highest cross-validated permutation importance among all investigated predictors. This apparent discrepancy suggests that simple linear associations alone are insufficient to characterize the complex mechanisms underlying hearing aid technology adaptation [5]. Instead, age appears to interact with demographic, behavioral, and clinical variables through nonlinear relationships captured by the ensemble learning model. This finding further illustrates the ability of Random Forest Regression to identify complex predictor interactions that conventional linear statistical approaches may fail to detect.

The cross-validated permutation feature importance analysis further demonstrated that onset year and education level ranked immediately after age in terms of predictive contribution, followed by hearing aid usage duration and daily hearing aid use. In contrast, gender, battery type, tinnitus, and vertigo contributed comparatively less to the predictive model. The relatively small variability of the permutation importance estimates across the five validation folds suggests that the ranking of predictor variables remained reasonably stable under the adopted validation strategy. From an audiological perspective, the relatively high importance assigned to hearing aid usage duration is consistent with the concept that sustained auditory stimulation and regular device use contribute to successful hearing aid adaptation over time [3]. Likewise, the contribution of educational level may reflect differences in health literacy, technology acceptance, communication strategies, and effective device management during the rehabilitation process. Overall, these findings indicate that hearing aid adaptation and satisfaction are influenced by multiple interacting demographic, behavioral, clinical, and device-related characteristics rather than by a single dominant predictor.

Consistent with the correlation analysis, the investigated predictor variables exhibited only negligible linear associations with total HATASS scores, indicating that conventional linear statistical analyses alone may not adequately describe the multidimensional nature of hearing aid satisfaction. The machine learning analyses further supported this observation, with Random Forest Regression consistently demonstrating the best overall predictive performance among the evaluated regression models under the adopted five-fold cross-validation framework. Although the achieved predictive performance remained modest, the improved performance of the ensemble learning model compared with conventional Linear Regression supports the presence of nonlinear relationships among the investigated variables [15]. Clinical outcomes in audiology are rarely determined by isolated variables; instead, they emerge from interactions among demographic characteristics, behavioral adaptation, clinical status, and hearing aid-related factors [10]. In this context, the explainable machine learning framework provided additional insights into the relative contributions of the investigated predictors beyond those obtainable with conventional statistical analyses alone [5]. The relatively low cross-validated coefficient of determination further suggests that the currently available demographic, clinical, behavioral, and hearing aid-related variables explain only a modest proportion of the variability in total HATASS scores. This finding emphasizes the inherently multidimensional nature of hearing aid satisfaction and suggests that additional clinical, psychosocial, and audiological factors not included in the present dataset may substantially influence patient-reported outcomes.

From the perspective of audiological practice, the proposed framework provides an interpretable analytical approach for investigating hearing aid technology adaptation and satisfaction. As illustrated in the proposed conceptual workflow, demographic, clinical, and hearing aid-related information can be integrated with explainable machine learning models to support predictive assessment and facilitate the interpretation of model outputs [16]. Rather than replacing conventional clinical assessment, the proposed framework is intended to complement existing evaluation procedures by providing additional information derived from data-driven analyses.

Moreover, as highlighted by Kehtarnavaz and Lobarinas (2025), the systematic incorporation of user preferences into hearing aid programming remains challenging because widely accepted standardized protocols are still lacking, often leading to individualized adjustments that are difficult to evaluate consistently in clinical practice [17]. In this context, the proposed framework represents an interpretable analytical approach that may support future investigations into more systematic, data-informed strategies for evaluating hearing aid technology adaptation and satisfaction.

The proposed workflow should not be interpreted as a fully autonomous clinical decision-making system. Instead, it is intended to function as a supportive analytical framework that complements clinician-centered decision-making during hearing aid rehabilitation and long-term user follow-up [18]. If validated in future longitudinal and multicenter studies, similar explainable predictive frameworks may assist clinicians in interpreting patient-specific outcomes while preserving the central role of clinical expertise in nonlinear ways through human–AI collaboration [13].

In a future validated implementation, such a framework would most plausibly be deployed within audiology clinics or hearing healthcare centers equipped with structured electronic health records and appropriate computational infrastructure. Its implementation would require collaboration among audiologists, clinical staff, and hearing aid technology specialists, rather than direct use by patients. The present study does not evaluate technical infrastructure, workflow integration, usability, economic feasibility, or clinical effectiveness. Consequently, the proposed framework should be regarded primarily as a research-oriented analytical prototype rather than a patient-facing clinical decision support tool.

Although five-fold cross-validation provides a substantially more reliable estimate of predictive performance than a single train–test split, the proposed framework has not yet been validated using independent external datasets. Therefore, the reported performance metrics should be interpreted as internally validated estimates rather than evidence of generalizable clinical performance. Future studies should incorporate repeated validation strategies, multicenter cohorts, and independent external validation datasets to further evaluate the robustness, reproducibility, and generalizability of the proposed framework across different clinical populations and healthcare settings.

One methodological strength of the present study is the use of the HATASS questionnaire, a multidimensional instrument specifically developed and psychometrically validated in Turkish. This instrument enables technology adaptation and satisfaction to be assessed using culturally and linguistically appropriate expressions, an important consideration in audiological rehabilitation where patient-reported experiences are inherently influenced by language and cultural context.

Overall, the findings of this study suggest that explainable machine learning can provide clinically interpretable insights into the multidimensional factors associated with hearing aid technology adaptation and satisfaction. Nevertheless, additional longitudinal investigations involving larger and more diverse study populations, objective audiological measurements, expanded psychosocial and clinical variables, and independent external validation remain necessary to improve predictive performance, external validity, and future clinical applicability across hearing healthcare settings [12,18].

### Limitations

Several limitations should be considered when interpreting the findings of this study. First, the dataset had a cross-sectional design, preventing the assessment of longitudinal changes in hearing aid technology adaptation and satisfaction over time. Consequently, the proposed framework captures associations within a single assessment period rather than temporal changes in patient outcomes.

Second, the dataset primarily consisted of self-reported questionnaire responses and subjective behavioral measures, including self-reported daily hearing aid usage duration. As reported in the audiological literature, self-reported hearing aid use may overestimate actual device usage when compared with objective data-logging measurements [19,20]. In addition, the predictor set did not include detailed audiological measurements, such as audiogram configurations, speech discrimination scores, hearing-loss severity, real-world hearing aid usage obtained from device data logging, or environmental acoustic classification [3]. Consequently, the proposed predictive framework was developed using demographic, clinical, and self-reported hearing aid-related variables rather than a more comprehensive set of objective audiological, physiological, and behavioral measurements.

Third, although model performance was evaluated using five-fold cross-validation, which provides a more reliable internal estimate of predictive performance than a single train–test partition, the proposed framework has not yet been validated on independent external datasets. Therefore, the reported performance metrics should be interpreted as internally validated estimates that may not generalize fully to other clinical populations, healthcare systems, or hearing aid technologies. Future studies should incorporate multicenter cohorts, repeated validation strategies, and independent external validation datasets to further evaluate the robustness and generalizability of the proposed framework.

Finally, the dataset exhibited a moderate ceiling effect, with HATASS scores tending to cluster toward the higher end of the scale. Although sufficient variability remained for regression-based modeling, the relatively modest predictive performance observed in the present study suggests that additional factors influencing hearing aid adaptation and satisfaction were not captured by the available predictors. Consistent with recent recommendations in medical artificial intelligence regarding dataset shift and model generalizability [10,18], future studies incorporating objective audiological measurements, longitudinal follow-up data, psychosocial variables, cognitive assessments, and broader clinical information may improve predictive performance, model robustness, and clinical applicability. Accordingly, the proposed HATASS-ML framework should be regarded as an interpretable research-oriented predictive framework that provides a foundation for future investigations rather than as a definitive clinical decision support system.

## 5. Conclusions

This study presents an explainable machine learning framework for investigating hearing aid technology adaptation and satisfaction using demographic, clinical, and hearing aid-related variables collected from adult hearing aid users. By combining comparative regression-based machine learning models with cross-validated explainability analysis, the proposed framework extends conventional statistical approaches and provides an interpretable perspective on the multidimensional factors associated with total HATASS scores.

The results demonstrate that hearing aid adaptation and satisfaction cannot be adequately characterized using isolated demographic variables or conventional linear statistical analyses alone. Although the initial correlation analysis revealed only negligible linear associations between the investigated predictors and total HATASS scores, the machine learning analyses identified meaningful differences in the relative contribution of individual variables. The cross-validated permutation importance analysis consistently identified age as the most influential predictor, followed by onset year, education level, hearing aid usage duration, and daily hearing aid use. These findings suggest that hearing aid satisfaction arises from complex nonlinear interactions among demographic, behavioral, clinical, and device-related characteristics rather than from any single dominant factor.

Among the evaluated regression algorithms, Random Forest Regression demonstrated the most consistent predictive performance within the adopted five-fold cross-validation framework, achieving the lowest prediction error and the highest coefficient of determination. Nevertheless, the relatively modest predictive performance observed across all evaluated algorithms indicates that the demographic, clinical, and hearing aid-related variables included in the present study explain only a limited proportion of the variability in total HATASS scores. This observation reinforces the inherently multidimensional nature of hearing aid technology adaptation and satisfaction and highlights the need to incorporate additional objective audiological, psychosocial, cognitive, and rehabilitation-related variables in future predictive models.

An important contribution of the present study is the integration of comparative machine learning, cross-validated permutation feature importance, and transparent model interpretation within a single analytical framework. Rather than functioning as a black-box prediction model, the proposed approach provides interpretable information regarding the relative contribution of individual predictors while maintaining an internally validated assessment of predictive performance. Such an approach may facilitate future research on explainable artificial intelligence in audiology and contribute to the development of more transparent data-driven decision support methodologies.

The proposed framework should not be interpreted as a fully autonomous clinical decision support system. Instead, it is intended as an interpretable research-oriented analytical tool that complements conventional audiological assessment by providing additional insights into the multidimensional factors associated with hearing aid technology adaptation and satisfaction. Although internal validation using five-fold cross-validation demonstrated consistent model behavior, external validation using independent multicenter datasets remains necessary before clinical implementation can be considered.

Future investigations should incorporate larger and more heterogeneous patient populations, longitudinal follow-up data, objective hearing aid data-logging measurements, cognitive and psychosocial assessments, and independent external validation cohorts. Integrating these complementary sources of information is expected to improve predictive performance, strengthen model generalizability, and further advance the application of explainable artificial intelligence for personalized hearing healthcare and hearing aid rehabilitation.

## Figures and Tables

**Table 1:** Descriptive statistics of the demographic variables and total HATASS scores.

Variable	Value
Sample Size	583
Age (years)	53.31 ± 16.97
Age Range (years)	18–87
Female	272 (46.66%)
Male	311 (53.34%)
Total HATASS Score	127.79 ± 15.55
Score Range	49–145

**Table 2:** Five-fold cross-validation performance of the investigated regression models for predicting the total HATASS score. Reported values represent the average RMSE, MAE, and coefficient of determination ($R^{2}$) across the five validation folds.

Model	RMSE	MAE	$R^{2}$
Linear Regression	15.612	11.548	−0.030
Decision Tree Regression	15.920	11.091	−0.077
Random Forest Regression	14.228	10.117	0.146
Support Vector Regression	15.200	9.758	0.020
Gradient Boosting Regression	17.359	9.246	−0.284

## Data Availability

The data presented in the main text and the Supplementary Material are available on request from the corresponding author. The data are not publicly available due to privacy and ethical restrictions.

## Supplementary Materials

The following supporting information can be downloaded at https://www.mdpi.com/article/10.3390/bioengineering13090985/s1.

## Abbreviations

The following abbreviations are used in this manuscript:

ANN	Artificial Neural Network
CDSS	Clinical Decision Support System
DF	Digital Functionality
DTR	Decision Tree Regression
GBR	Gradient Boosting Regression
HATASS	Hearing Aid Technology Adaptation and Satisfaction Scale
LR	Linear Regression
MAE	Mean Absolute Error
ML	Machine Learning
$R^{2}$	Coefficient of Determination
RFR	Random Forest Regression
RMSE	Root Mean Square Error
SVR	Support Vector Regression
TSE	Technological Satisfaction Experience
UD	Usage Difficulty
XAI	Explainable Artificial Intelligence
