# Supplementary material for: An Explainable Machine Learning Framework for Predicting Hearing Aid Satisfaction: Integrating the HATASS Instrument and Clinical Insights
Source: Bioengineering (Basel). 2026 Aug 26;13(9):985. doi: 10.3390/bioengineering13090985 (PMC13603878; doi:10.3390/bioengineering13090985)
Supplement: Supplementary file 1 [file bioengineering-13-00985-s001.zip › bioengineering-4503196-supplementary.pdf]

HATASS Instrument Content

Appendix 1. Final HATASS administration form.

Aşağıda yer alan ifadeler, işitme cihazlarınız hakkındaki düşüncelerinizi değerlendirmek amacıyla hazırlanmıştır. Lütfen her bir ifadeyi dikkatlice okuyunuz ve sizin için en uygun seçeneği işaretleyiniz. Yanıt seçenekleri aşağıda belirtilmiştir. Vereceğiniz yanıtların, şu anda kullanmakta olduğunuz ya da son zamanlarda en sık kullandığınız işitme cihazına ilişkin genel deneyimlerinizi yansıtması beklenmektedir.

**Madde No**	**İfade**	**Kesinlikle Katılmıyorum**	**Katılmıyorum**	**Kararsızım**	**Katılıyorum**	**Kesinlikle Katılıyorum**
**1.**	İşitme cihazı kullanımı yaşam standartlarımı (özel hayat, iş hayatı, sosyal hayat vb.) olumlu yönde etkiler.					
**2.**	İşitme cihazımın kullanımı rahat ve pratik.					
**3.**	İşitme cihazımın teknolojik özellikleri iletişim yeteneklerimi olumlu yönde etkiler.					
**4.**	Beklenmedik yüksek sesleri (yangın dedektörü, ambulans sireni vb.) duyduğumda rahatsız olurum.					
**5.**	İşitme cihazımın dijital özelliklerinden memnunum.					
**6.**	Cihazımın sessiz ortamlardaki (sessiz bir odada iletişim kurmak vb.) performansından memnunum.					
**7.**	İşitme cihazımın kablosuz bağlantı özelliği ile müzik/radyo dinlemek, video izlemek, telefonla sesli veya görüntülü konuşma gibi aktiviteleri gerçekleştirmek yaşamımı kolaylaştırıyor.					
**8.**	Yanımda yedek pil veya şarj aleti taşımakta zorlanıyorum.					
**9.**	Sinema, tiyatro ve konferans gibi ortamlarda insanların fısıldaşarak yaptığı konuşmaları duymak beni memnun eder.					
**10.**	İşitme cihazımı mobil uygulama üzerinden yönetmek (ses ayarı yapabilme, uzaktan klinisyenle görüşme ve uzaktan ayar, cihazımı bul yardımı vb.) kullanım kolaylığı sağlıyor.					

**11.**	İşitme cihazımı taktığımda işitme kaybının olumsuz etkilerini (iletişim problemi, söylenenleri anlayamama vb.) yaşamam.					
**12.**	Cihazım takılıyken kendi sesimi rahatsız edici şekilde duyarım.					
**13.**	İşitme cihazım ses aktarımı sırasında (TV, Bluetooth, tele-coil, telefon bağlantısı vb.) sesleri doğal ve net bir şekilde duymamı sağlar.					
**14.**	Cihazımın gürültülü ortamlardaki (kalabalık ortamlar, trafik, inşaat gürültüsü, konser vb.) performansından memnunum.					
**15.**	İşitme cihazımı kullandığım durumlarda söylenenleri daha az tekrar ettiririm.					
**16.**	Cihazımın dış görünüşünden rahatsızlık duyarım.					
**17.**	İşitme cihazım sosyal hayatta kaygı yaşamama (yetersizlik hissi, damgalanma korkusu vb.) engel olur.					
**18.**	Çok fonksiyonlu düğmenin (ses ayarı/program değişimi yapmak, aramaları cevaplamak vb.) işlevselliğini yeterli buluyorum.					

Lütfen aşağıda belirtilen özellikleri dikkate alarak "İşitme cihazımın sunduğu özellikler günlük yaşantıdaki ihtiyaçlarımı karşılıyor." ifadesine katılım düzeyinizi işaretleyiniz.

**Madde No**	**İfade**	**Kesinlikle Katılmıyorum**	**Katılmıyorum**	**Kararsızım**	**Katılıyorum**	**Kesinlikle Katılıyorum**
**1.**	Mikrofon yönü (tüm yönlerden gelen sesleri) ayarlama					
**2.**	Ortam ve program seçimi					
**3.**	Otomatik ses aktarım modu					
**4.**	Aksesuarlar					
**5.**	Kontakt (temas) gürültü önleyici]					
**6.**	Ötme kontrol sistemi					
**7.**	Gürültü azaltma					
**8.**	Rüzgar sesi bastırma					
**9.**	Yankı önleyici					
**10.**	Pil göstergesi					
**11.**	Tinnitus yardımı					

Appendix 2. English version of HATASS organized by subdimensions.

The English version of the scale was developed through forward translation and expert review to ensure conceptual equivalence with the original Turkish version.

THREE DIMENSIONS	ITEMS	
TECHNOLOGICAL SATISFACTION EXPERIENCE (TSE)	I1	Using my hearing aid has a positive impact on my quality of life (personal life, professional life, social life, etc.).
	I2	My hearing aid is comfortable and practical to use.
	I3	The technological features of my hearing aid have a positive impact on my ability to communicate.
	I4	I am satisfied with the digital features of my hearing aid.
	I5	I am satisfied with the performance of my hearing aid in quiet environments (e.g., communicating in a quiet room).
	I6	The wireless connectivity feature of my hearing aid makes my life easier by allowing me to listen to music or the radio, watch videos, and make voice or video calls on my phone.
	I7	I am satisfied when I am able to hear whispered conversations in environments such as cinemas, theaters, and conferences.
	I8	Managing my hearing aid through a mobile application (e.g., adjusting volume settings, remote consultations with a clinician, remote adjustments, and the “Find My Device” feature) makes it easier to use.
	I9	When I wear my hearing aid, I do not experience the negative effects of hearing loss (e.g., communication difficulties and difficulty understanding speech).
	I10	My hearing aid allows me to hear sounds naturally and clearly during audio streaming activities (e.g., TV, Bluetooth, telecoil, and phone connectivity).
	I11	I am satisfied with the performance of my hearing aid in noisy environments (e.g., crowded places, traffic, construction noise, and concerts).
	I12	When I use my hearing aid, I ask people to repeat themselves less often.
	I13	My hearing aid prevents me from experiencing anxiety in social situations (e.g., feelings of inadequacy and fear of stigma).
	I14	I find the functionality of the multifunction button (e.g., adjusting the volume, changing programs, and answering calls) to be adequate.
DIGITAL FUNCTIONALITY (DF)	I15	The features provided by my hearing aid meet my daily needs. [Adjusting microphone directionality (sounds coming from all directions)]
	I16	The features provided by my hearing aid meet my daily needs. [Environment and program selection]
	I17	The features provided by my hearing aid meet my daily needs. [Automatic streaming mode]
	I18	The features provided by my hearing aid meet my daily needs. [Accessories]
	I19	The features provided by my hearing aid meet my daily needs. [Contact noise reduction]
	I20	The features provided by my hearing aid meet my daily needs. [Feedback control system]
	I21	The features provided by my hearing aid meet my daily needs. [Noise reduction]
	I22	The features provided by my hearing aid meet my daily needs. [Wind noise reduction]
	I23	The features provided by my hearing aid meet my daily needs. [Echo reduction]
	I24	The features provided by my hearing aid meet my daily needs. [Battery indicator]
	I25	The features provided by my hearing aid meet my daily needs. [Tinnitus support]
USAGE DIFFICULTY (UD)	I26	I feel uncomfortable when I hear unexpected loud sounds (e.g., fire alarms, ambulance sirens).
	I27	I find it difficult to carry a spare battery or charger with me.
	I28	I hear my own voice in an uncomfortable way when wearing my hearing aid.
	I29	I feel uncomfortable with the appearance of my hearing aid.

Appendix 3. Original Turkish version of HATASS organized by subdimensions.
The original Turkish version of HATASS is provided for reference.

**3 BOYUT**		İFADELER
TEKNOLOJİK MEMNUNİYET DENEYİMİ	M1	İşitme cihazı kullanımı yaşam standartlarımı (özel hayat, iş hayatı, sosyal hayat vb.) olumlu yönde etkiler.
	M2	İşitme cihazımın kullanımı rahat ve pratik.
	M3	İşitme cihazımın teknolojik özellikleri iletişim yeteneklerimi olumlu yönde etkiler.
	M4	İşitme cihazımın dijital özelliklerinden memnunum.
	M5	Cihazımın sessiz ortamlardaki (sessiz bir odada iletişim kurmak vb.) performansından memnunum.
	M6	İşitme cihazımın kablosuz bağlantı özelliği ile müzik/radyo dinlemek, video izlemek, telefonla sesli veya görüntülü konuşma gibi aktiviteleri gerçekleştirmek yaşamımı kolaylaştırıyor.
	M7	Sinema, tiyatro ve konferans gibi ortamlarda insanların fısıldaşarak yaptığı konuşmaları duymak beni memnun eder.
	M8	İşitme cihazımı mobil uygulama üzerinden yönetmek (ses ayarı yapabilme, uzaktan klinisyenle görüşme ve uzaktan ayar, cihazımı bul yardımı vb.) kullanım kolaylığı sağlıyor.
	M9	İşitme cihazımı taktığımda işitme kaybının olumsuz etkilerini (iletişim problemi, söylenenleri anlayamama vb.) yaşamam.
	M10	İşitme cihazım ses aktarımı sırasında (TV, Bluetooth, tele-coil, telefon bağlantısı vb.) sesleri doğal ve net bir şekilde duymamı sağlar.
	M11	Cihazımın gürültülü ortamlardaki (kalabalık ortamlar, trafik, inşaat gürültüsü, konser vb.) performansından memnunum.
	M12	İşitme cihazımı kullandığım durumlarda söylenenleri daha az tekrar ettiririm.
	M13	İşitme cihazım sosyal hayatta kaygı yaşamama (yetersizlik hissi, damgalanma korkusu vb.) engel olur.
	M14	Çok fonksiyonlu düğmenin (ses ayarı/program değişimi yapmak, aramaları cevaplamak vb.) işlevselliğini yeterli buluyorum.
DİJİTAL İŞLEVSELLİK	M15	İşitme cihazımın sunduğu özellikler günlük yaşantıdaki ihtiyaçlarımı karşılıyor. [Mikrofon yönü (tüm yönlerden gelen sesleri) ayarlama]
	M16	İşitme cihazımın sunduğu özellikler günlük yaşantıdaki ihtiyaçlarımı karşılıyor. [Ortam ve program seçimi]
	M17	İşitme cihazımın sunduğu özellikler günlük yaşantıdaki ihtiyaçlarımı karşılıyor. [Otomatik ses aktarım modu]
	M18	İşitme cihazımın sunduğu özellikler günlük yaşantıdaki ihtiyaçlarımı karşılıyor. [Aksesuarlar]
	M19	İşitme cihazımın sunduğu özellikler günlük yaşantıdaki ihtiyaçlarımı karşılıyor. [Kontakt (temas) gürültü önleyici]
	M20	İşitme cihazımın sunduğu özellikler günlük yaşantıdaki ihtiyaçlarımı karşılıyor. [Ötme kontrol sistemi]
	M21	İşitme cihazımın sunduğu özellikler günlük yaşantıdaki ihtiyaçlarımı karşılıyor. [Gürültü azaltma]
	M22	İşitme cihazımın sunduğu özellikler günlük yaşantıdaki ihtiyaçlarımı karşılıyor. [Rüzgar sesi bastırma]
	M23	İşitme cihazımın sunduğu özellikler günlük yaşantıdaki ihtiyaçlarımı karşılıyor. [Yankı önleyici]
	M24	İşitme cihazımın sunduğu özellikler günlük yaşantıdaki ihtiyaçlarımı karşılıyor. [Pil göstergesi]
	M25	İşitme cihazımın sunduğu özellikler günlük yaşantıdaki ihtiyaçlarımı karşılıyor. [Tinnitus yardımı]
KULLANIM ZORLUĞU	M26	Beklenmedik yüksek sesleri (yangın dedektörü, ambulans sireni vb.) duyduğumda rahatsız olurum.
	M27	Yanımda yedek pil veya şarj aleti taşımakta zorlanıyorum.
	M28	Cihazım takılıyken kendi sesimi rahatsız edici şekilde duyarım.
	M29	Cihazımın dış görünüşünden rahatsızlık duyarım.

HATASS Psychometric Validation Results

Table S1. Descriptive statistics of the EFA sample (*n* = 202).

**Categorical Variables**	**Options**		***n***	**%**
Gender	Female		94	46.5
	Male		108	53.5
Hearing Aid Power Source	Battery-powered		175	86.6
	Rechargeable		27	13.4
	Right		64	31.7
Side of Hearing Loss	Left		39	19.3
	Bilateral		99	49.0
Tinnitus	Yes		60	29.7
	No		142	70.3
Persistent Dizziness	Yes		19	9.4
	No		183	90.6
Education Level	Primary School		41	20.3
	Middle School		33	16.3
	High School		63	31.2
	University		65	32.2
**Continuous Variables**	***M***	***SD***	**Min.**	**Max.**
Age (years)	51.08	17.13	18	85
Duration of Hearing Loss (years)	5.96	8.33	1	50
Duration of Hearing Aid Use (years)	4.27	5.95	1	38
Daily Hearing Aid Use (hours)	12.24	2.90	3	19

Note. M = mean; *SD* = standard deviation.

Table S2. EFA results of HATASS.

**Factors and items**	**Eigenvalue**	**Explained variance (%)**	*M*	*SD*	Item–total correlation	Factor loading
Factor 1 (TSE) (α = .968)						
I29			4.62	0.78	.682	0.893
I16			4.54	0.83	.719	0.879
I32			4.50	0.79	.686	0.874
I14			4.49	0.85	.731	0.874
I19			4.52	0.92	.697	0.871
I24			4.46	0.88	.673	0.838
I20	14.374	49.56	4.45	0.95	.633	0.801
I1			4.43	0.93	.737	0.799
I31			4.54	0.88	.697	0.785
I2			4.37	0.94	.614	0.758
I18			4.35	1.03	.744	0.750
I34			4.49	0.97	.690	0.748
I30			4.44	1.03	.619	0.738
I37			4.31	0.91	.537	0.732
Factor 2 (DF) (α = .969)						
I7			4.47	0.92	.762	0.875
I9			4.43	0.95	.776	0.869
I8			4.46	0.93	.755	0.861
I12			4.37	1.00	.744	0.849
I5			4.43	0.95	.802	0.847
I10	4.855	16.74	4.44	0.90	.736	0.845
I3			4.39	0.92	.771	0.842
I4			4.38	0.96	.776	0.833
I6			4.38	0.92	.751	0.824
I11			4.43	0.96	.703	0.824
I13			4.11	1.12	.605	0.726
Factor 3 (UD) (α = .828)						
I22			3.51	1.54	.148	0.848
I36	2.103	7.25	3.51	1.50	.121	0.843
I21			3.93	1.37	.130	0.809
I33			3.92	1.26	.155	0.683

Note. TSE = technological satisfaction experience; DF = digital functionality; UD = usage difficulty; I = item; n = 202; M = mean; SD = standard deviation; overall Cronbach's α = .942; total variance explained = 73.56%; KMO = .941; $\chi^2(406) = 6377.478$, $p < .001$.

Table S3. Descriptive statistics of the CFA sample (n = 381).

**Categorical Variables**	**Options**		***n***	**%**
Gender	Female		178	46.7
	Male		203	53.3
Hearing Aid Power Source	Battery-powered		302	79.3
	Rechargeable		79	20.7
	Right		89	23.4
Side of Hearing Loss	Left		73	19.2
	Bilateral		219	57.5
Tinnitus	Yes		91	23.9
	No		290	76.1
Persistent Dizziness	Yes		28	7.3
	No		353	92.7
Education Level	Primary School		97	25.5
	Middle School		79	20.7
	High School		103	27.0
	University		102	26.8
**Continuous Variables**	***M***	***SD***	**Min.**	**Max.**
Age (years)	54.50	16.79	18	87
Duration of Hearing Loss (years)	5.88	8.31	1	71
Duration of Hearing Aid Use (years)	3.49	4.56	1	38
Daily Hearing Aid Use (hours)	12.16	2.75	3	19

Note. M = mean; SD = standard deviation.

Table S4. Model fit indices for HATASS.

**Goodness-of-Fit Index**	**Model Value**	**Acceptable Fit**	**Good Fit**
χ^2/df	2.85	$2 \leq \chi^2/df \leq 5$	$0 \leq \chi^2/df \leq 2$
RMSEA	.079	.05 ≤ RMSEA ≤ .08	0 ≤ RMSEA ≤ .05
RMR	.035	.05 ≤ RMR ≤ .08	0 ≤ RMR ≤ .05
NFI	.92	.90 ≤ NFI ≤ .95	.95 ≤ NFI ≤ 1.00
TLI	.93	.90 ≤ NNFI ≤ .95	.95 ≤ NNFI ≤ 1.00
CFI	.94	.90 ≤ CFI ≤ .95	.95 ≤ CFI ≤ 1.00
RFI	.91	.90 ≤ RFI ≤ .95	.95 ≤RFI ≤ 1.00
GFI	.88	.85 ≤ GFI ≤ .90	.90 ≤ GFI ≤ 1.00
IFI	.94	.90 ≤ IFI ≤ .95	.95 ≤ IFI ≤ 1.00

Note. χ^2 = 401.30; df = 141.

Table S5. Construct reliability and convergent validity findings for HATASS.

Factor	Number of Items	CR	AVE
Technological Satisfaction Experience	14	.98	.78
Digital Functionality	11	.97	.76
Usage Difficulty	4	.86	.60

Table S6. Discriminant validity results according to the Fornell–Larcker criterion.

Factor	DF	TSE	UD
**DF**	**.87***	.41	.17
**TSE**		**.88***	-.25
**UD**			**.77***

Note. *** Diagonal values represent the √AVE values for each factor, whereas off-diagonal values represent inter-factor correlations;** TSE = technological satisfaction experience; DF = digital functionality; UD = usage difficulty.

Table S7. Internal consistency (Cronbach's alpha) coefficients of HATASS.

Factors	Cronbach's Alpha
Technological Satisfaction Experience	.975
Digital Functionality	.970
Usage Difficulty	.852
Overall HATASS Alpha	.914

Note. HATASS = Hearing Aid Technology Adaptation and Satisfaction Scale.

Table S8. Comparison of HATASS items between the lower and upper 27% groups.

Factors		Lower 27% (*n*=103)		Upper 27% (*n*=103)		*t*	*p*
		M	*SD*	*M*	*SD*		
Technological Satisfaction Experience	I29	4.20	1.00	4.99	.10	-7.915	**.000***
	I16	4.14	0.98	4.98	.14	-8.655	**.000***
	I32	4.04	1.02	4.96	.19	-9.027	**.000***
	I14	4.15	1.00	4.99	.10	-8.497	**.000***
	I19	4.12	0.99	4.98	.14	-8.745	**.000***
	I24	3.99	1.03	4.97	.17	-9.501	**.000***
	I20	4.04	1.01	4.94	.24	-8.844	**.000***
	I1	4.06	1.03	4.97	.17	-8.896	**.000***
	I31	4.04	0.99	4.98	.20	-9.474	**.000***
	I2	3.95	1.04	4.95	.22	-9.537	**.000***
	I18	3.97	0.98	4.99	.10	-10.454	**.000***
	I34	4.12	0.95	4.89	.52	-7.255	**.000***
	I30	3.91	1.25	4.77	.94	-5.532	**.000***
	I37	3.98	1.04	4.85	.45	-7.832	**.000***
Digital Functionality	I7	4.00	0.75	4.97	.17	-12.751	**.000***
	I9	3.95	0.81	4.99	.10	-12.937	**.000***
	I8	3.88	0.83	4.98	.14	-13.201	**.000***
	I12	3.93	0.82	4.93	.25	-11.832	**.000***
	I5	4.01	0.87	5.00	.00	-11.568	**.000***
	I10	3.94	0.83	4.98	.14	-12.583	**.000***
	I3	4.01	0.82	4.99	.10	-12.015	**.000***
	I4	3.95	0.82	4.97	.22	-12.174	**.000***
	I6	3.97	0.83	4.99	.10	-12.322	**.000***
	I11	3.94	0.84	4.99	.10	-12.610	**.000***
	I13	3.67	0.84	4.68	.70	-9.324	**.000***
Usage Difficulty	I22	2.78	1.47	4.40	1.01	-9.225	**.000***
	I36	2.76	1.40	4.61	.53	-12.604	**.000***
	I21	3.20	1.59	4.60	.72	-8.121	**.000***
	I33	3.15	1.40	4.69	.47	-10.599	**.000***

Note. *M* = mean; *SD* = standard deviation; * $p \leq .05$; ** $p \leq .01$; *** $p \leq .001$.

Table S9. Pearson correlation coefficients between the HATASS test–retest measurements.

	TSE		DF		UD		Overall HATASS	
	r	***p***	***r***	***p***	***r***	***p***	***r***	***p***
**TSE**	.996	.000***						
**DF**			.992	.000***				
**UD**					.982	.000***		
**Overall HATASS**							.992	.000***

Note. *** = $p \leq .001$; TSE = technological satisfaction experience; DF = digital functionality; UD = usage difficulty; HATASS = Hearing Aid Technology Adaptation and Satisfaction Scale.

Table S10. Independent samples t-test results.

			n	*M*	*SD*	*t*	*p*
Hearing Aid Power Source	TSE	Battery-powered	302	63.92	9.41	-0.302	0.763
		Rechargeable	79	64.27	7.94		
	DF	Battery-powered	302	50.97	6.61	1.172	0.242
		Rechargeable	79	50.00	6.42		
	UD	Battery-powered	302	10.33	5.37	3.838	**.000*****
		Rechargeable	79	7.89	3.59		
	Overall HATASS	Battery-powered	302	128.55	13.95	-1.044	0.297
		Rechargeable	79	130.38	13.44		
Tinnitus	TSE	Yes	91	62.20	12.41	-1.708	0.090
		No	290	64.55	7.75		
	DF	Yes	91	50.40	7.24	-0.584	0.560
		No	290	50.89	6.36		
	UD	Yes	91	8.02	4.07	-3.912	**.000*****
		No	290	10.39	5.32		
	Overall HATASS	Yes	91	128.57	17.94	-0.235	0.815
		No	290	129.04	12.33		
Persistent Dizziness	TSE	Yes	28	62.50	10.70	-0.898	0.370
		No	353	64.11	8.99		
	DF	Yes	28	49.46	7.05	-1.093	0.275
		No	353	50.88	6.54		
	UD	Yes	28	8.68	4.78	-1.230	0.219
		No	353	9.92	5.17		
	Overall HATASS	Yes	28	127.29	17.70	-0.653	0.514
		No	353	129.06	13.52		

Note. TSE = technological satisfaction experience; DF = digital functionality; UD = usage difficulty; HATASS = Hearing Aid Technology Adaptation and Satisfaction Scale; *M* = mean; *SD* = standard deviation; *** = $p \leq .001$.

Table S11. One-way ANOVA results.

			n	*M*	*SD*	*F*	*p*	**Post Hoc Comparison**
Side of Hearing Loss	TSE	1. Right	89	64.34	9.64	0.144	0.866	
		2. Left	73	63.56	11.18			
		3. Bilateral	219	63.99	8.12			
	DF	1. Right	89	51.57	6.42	0.912	0.403	
		2. Left	73	50.74	6.43			
		3. Bilateral	219	50.46	6.69			
	UD	1. Right	89	9.39	4.77	.486	0.616	
		2. Left	73	9.77	5.48			
		3. Bilateral	219	10.03	5.19			
	Overall HATASS	1. Right	89	130.52	13.78	0.761	0.468	
		2. Left	73	128.53	15.25			
		3. Bilateral	219	128.42	13.40			
Education Level	TSE	1. Primary School	97	63.86	8.55	0.440	0.725	
		2. Middle School	79	65.00	7.76			
		3. High School	103	63.50	9.41			
		4. University	102	63.83	10.30			
	DF	1. Primary School	97	51.14	6.02	0.895	0.444	
		2. Middle School	79	49.89	6.47			
		3. High School	103	51.35	5.83			
		4. University	102	50.52	7.78			
	UD	1. Primary School	97	11.97	5.56	21.560	**.000*****	
		2. Middle School	79	11.84	5.50			**3-4 < 1**
		3. High School	103	8.57	3.95			**3-4 < 2**
		4. University	102	7.51	4.07			
	Overall HATASS	1. Primary School	97	127.03	11.91	2.081	0.102	
		2. Middle School	79	127.05	12.65			
		3. High School	103	130.27	14.62			
		4. University	102	130.84	15.36			

Note. TSE = technological satisfaction experience; DF = digital functionality; UD = usage difficulty; HATASS = Hearing Aid Technology Adaptation and Satisfaction Scale; *M* = mean; *SD* = standard deviation; *** = $p \leq .001$.

Table S12. Pearson correlations between HATASS scores and participant variables.

		TSE	DF	UD	Overall HATASS
Age (years)	*r*	**.118***	**.135****	**.220****	.060
	p	0.021	0.008	0.000	0.243
	n	381	381	381	381
Duration of Hearing Loss (years)	*r*	-.046	.033	-.028	-.004
	p	0.371	0.521	0.586	0.935
	n	381	381	381	381
Duration of Hearing Aid Use (years)	*r*	-.031	-.004	-.079	.007
	p	0.549	0.932	0.125	0.893
	n	381	381	381	381
Daily Hearing Aid Use (hours)	*r*	.004	-.012	-.023	.005
	p	0.944	0.817	0.650	0.916
	n	381	381	381	381

Note. TSE = technological satisfaction experience; DF = digital functionality; UD = usage difficulty; HATASS = Hearing Aid Technology Adaptation and Satisfaction Scale; * $p \leq .05$; ** $p \leq .01$; *** $p \leq .001$.
